# Increasing the shockable rhythm and survival rate by dispatcher-assisted cardiopulmonary resuscitation in Japan

**DOI:** 10.1016/j.resplu.2021.100122

**Published:** 2021-04-24

**Authors:** Minoru Kayanuma, Ryo Sagisaka, Hideharu Tanaka, Shota Tanaka

**Affiliations:** a Fujigoko Fire Department, Yamanashi, Japan; b Graduate School of Emergency Medical System, Kokushikan University, Tokyo, Japan; c Department of Integrated Science and Engineering for Sustainable Society, Chuo University, Tokyo, Japan; d Research Institute of Disaster Management and Emergency Medical System, Kokushikan University, Tokyo, Japan; e Tokai University School of Medicine, Kanagawa, Japan

**Keywords:** Out-of-hospital cardiac arrest, Bystander cardiopulmonary resuscitation, Dispatcher

## Abstract

**Purpose:**

This study aimed to examine the effectiveness of cardiopulmonary resuscitation (CPR) directions by dispatchers. We analysed the relationship of dispatcher-assisted bystander cardiopulmonary resuscitation (DA-BCPR) with favourable cerebral function, shockable rhythm rate, and emergency medical service (EMS) arrival time.

**Methods:**

This nationwide study was based on CPR statistical data of out-of-hospital cardiac arrest (OHCA) patients (n = 629,471) from 1 January 2011 to 31 December 2015, and included 107,669 patients with bystander-witnessed cardiogenic cardiac arrest.

The primary outcome was good brain function prognosis after 1 month, while the secondary outcome was the rate of shockable rhythm on ECG at the time of EMS arrival.

EMS arrival time at the site was stratified into 7 min, 8−10 min, and 11−20 min using tertiles. Adjusted odds ratios (AORs) and 95% confidence intervals (95% CIs) were estimated using multivariate logistic regression analysis to assess the association between DA-BCPR and outcomes in each tertile.

**Results:**

There were 37,269 (35%), 18,109 (17%), and 52,291 (49%) patients in the DA-BCPR, Only-BCPR, and no-BCPR groups, respectively. Compared to No-BCPR, DA-BCPR was associated with favourable neurological outcomes regardless of the time from 119 call to EMS contact (AOR, 1.56, 2.01, 1.82; 95% CI, 1.43−1.71, 1.80–2.24, 1.52−2.19; ≤7 min, 8–10 min, and 11–20 min, respectively). DA-BCPR showed association with the shockable rhythm rate upon EMS arrival regardless of the time 119 call to EMS contact (AOR, 1.30, 1.60, 1.90; 95% CI, 1.23−1.38, 1.51−1.70, 1.75–2.06; ≤7 min, 8–10 min, and 11–20 min, respectively).

**Conclusion:**

Providing dispatcher assistance with CPR to 119 callers improves the long-term outcome regardless of the patient’s age and EMS response time. Thus, encouraging dispatchers to promote BCPR is important for increasing the shockable rhythm rate and improving the brain function prognosis.

## Introduction

Out-of-hospital cardiac arrest (OHCA) is internationally recognized as a significant public health problem.^1^, ^2^, ^3^, ^4^ The post cardiac arrest survival rate is low even in high-income countries.^5^ In Japan, 126,000 OHCA cases occur annually. Although the one-month survival rate has improved, the survival rate and neurological outcome at one month in citizen-witnessed cardiogenic OHCA cases are still as low as 13.0% and 8.6%, respectively.^6^

In order to achieve return of spontaneous circulation (ROSC) and improve the neurological function in cardiogenic OHCA cases, bystanders may apply an automated external defibrillator (AED). Early bystander cardiopulmonary resuscitation (BCPR) has been reported to be significantly effective.^7^, ^8^, ^9^, ^10^, ^11^, ^12^, ^13^, ^14^, ^15^, ^16^, ^17^

The time to start CPR is crucial for the survival of the victim.^18^, ^19^ In Japan, since 1999, dispatchers from the fire department have helped citizen rescuers to recognise cardiac arrest at the 119-call stage, and have encouraged dispatcher assisted (DA) cardiopulmonary resuscitation (CPR) early after cardiac arrest. The importance of this system has also been emphasised in the Japanese version of the 2015 Japanese Resuscitation Council Guidelines.^20^ In a Japanese nationwide observational study on cardiogenic OHCA, cases where DA-BCPR was performed showed a higher improvement in the neurological outcome compared to cases without DA-BCPR. This effect was similar to that of spontaneously performed BCPR. Cerebral function outcome at one month correlated with the improvement in the shockable rhythm rate upon arrival of the emergency medical service (EMS).^21^ Several studies from outside of Japan have shown that dispatcher assistance in cardiogenic OHCA cases correlates with long-term outcome improvement.^22^, ^23^, ^24^

Consequently, early DA-BCPR is crucial for improving the outcomes of cardiac arrest patients. Early EMS intervention can also lead to improved outcome.^25^ However, in some areas, early EMS intervention cannot be provided in OHCA cases. The average time between an emergency 119 call and EMS arrival on the scene was 7.1 min in the shortest prefecture and 10.9 min in the longest prefecture (3.8 min difference).^6^ The purpose of this study was to analyse the correlation between DA-BCPR and the EMS arrival time and shockable rhythm rate, which can affect the neurological and long-term outcomes.

## Methods

### Study design

This nationwide, population-based cohort study used the Utstein database in Japan and was approved by the Institutional Review Board of Kokushikan University (#18013).

### Japanese EMS and dispatch systems

In Japan, in the event of a fire, emergency, or rescue situation, a common emergency number (119) is used to notify the nearest fire department (FD). As of 2016, there were 733 FDs in Japan, and the employees working at the dispatch centre at each FD receive the 119 calls and provide DA if needed. The Fire and Disaster Management Agency (FDMA) of the Ministry of Internal Affairs and Communications oversees the emergency medical service (EMS) agencies nationwide. Additionally, local fire departments operate on-site EMS systems, and a group of three people operates the EMS for one injured person. Generally, Japanese ambulances heading to the emergency scene do not have a doctor on board. Advanced life support, advanced airway management, venous access, and defibrillation with a semi-automatic defibrillator are performed by on-board paramedics. Professionally trained and certified paramedics can administer adrenaline (to 15 years or older individuals) and perform tracheal intubation with online instructions from a doctor. The medical control (MC) system ensures the quality of the first aid given by the paramedics between the request for emergency assistance and transfer to a medical institution. All first-aid protocols are validated and controlled. The DA standard was first presented by the FDMA in 1999. Subsequently, notifications were issued in 2005, 2013, and 2016 to strengthen the implementation at each fire department. After the 2013 notification, verification by doctors and specific education of the dispatchers began under the control of the MC system, and improvements were made in stages.^6^ If the dispatchers, who are the contact persons at 119, determine that first aid is necessary based on the content of the call, they verbally instruct the callers on the method of first aid over the telephone. From 2013, dispatchers have been giving DA in life-threatening cases, such as by assisting implementation of cardiopulmonary resuscitation involving only chest compressions.

### Data collection/Quality control

The Utstein style was used for the OHCA registration database. The Utstein database began in 2005 as an official registration. The FDMA manages the data. The following parameters were recorded: sex, age, witnessed or not, type of bystander, BCPR or not, public access defibrillation, DA, initial ECG upon EMS arrival, EMS intervention (defibrillation, intravenous line, adrenaline administration, use of airway management), time course of resuscitation, pre-hospital ROSC, cause of cardiopulmonary arrest, survival at one-month, and cerebral neurological outcome at one month. The Cerebral Performance Category (CPC) scale was used to evaluate cerebral neurological outcomes: category 1, good cerebral performance; category 2, moderate cerebral disability; category 3, severe cerebral disability; category 4, vegetative state or coma; and category 5, death. This evaluation was conducted by an emergency medical physician.

### Inclusion criteria

In Japan, oral guidance was provided in 1999, but it was not sufficiently unified and communicated to more than 700 firefighting organisations nationwide. In the 2010 Resuscitation Guidelines, the importance of oral guidance was announced, and it was thoroughly implemented by firefighting organisations nationwide from the beginning of 2011. Therefore, this study covers the five-year period from 2011 to 2015.

**Fig. 1:**
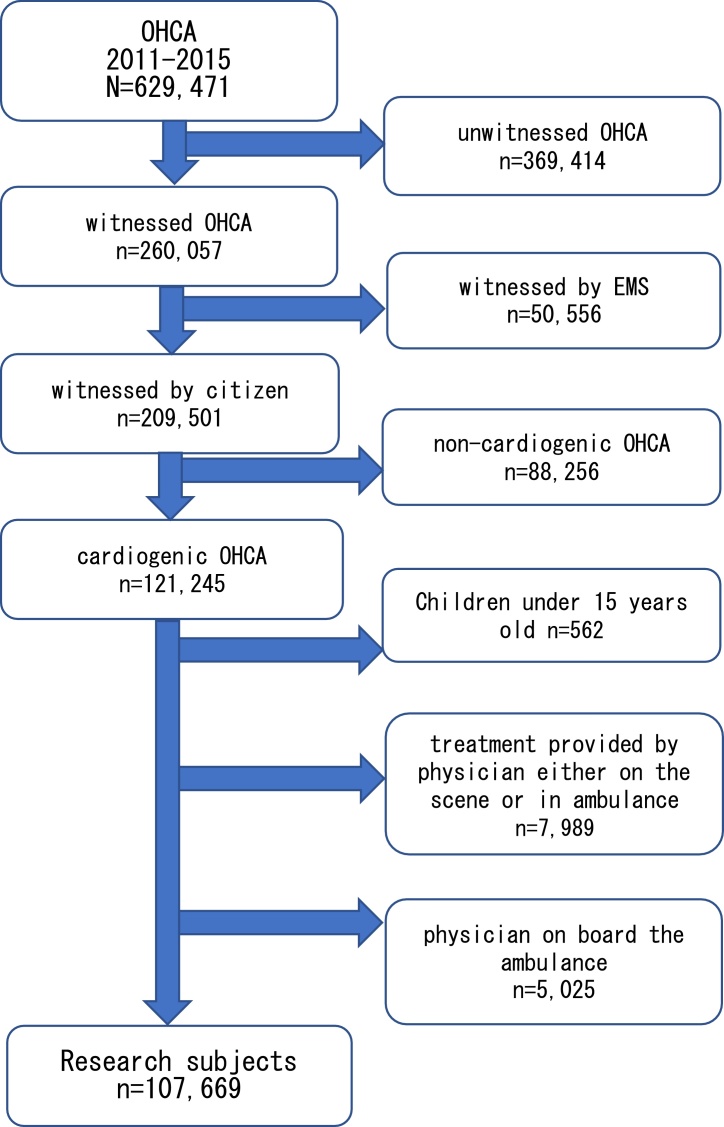
Extraction conditions for research subjects.

We included OHCA data from the FDMA between 1 January 2011 and 31 December 2015. The inclusion criteria were as follows: 1) age between 15 and 114 years, 2) witnessed by a citizen, and 3) cardiogenic OHCA. The exclusion criteria were 1) doctor on board the ambulance, 2) treatment by a doctor in the field and ambulance, and 3) children under 15 years of age (Fig. 1). Regarding the definition of exclusion criteria 1) and 2), doctors rarely ride in ambulances in Japan, and in rare cases where a doctor is present at the site, the doctor arrives at the site in another vehicle. In case the doctor arrives by medical helicopter, the doctor may reach the scene first, and may be already present at the scene. Such cases were excluded because there was no data regarding the drugs administered by the doctor, or the treatment given in the ambulance or in the field. Regarding 3), under 15 years old is defined as a child in Japan, and the age at which consent is valid is 15 years and over. Therefore, we excluded those under the age of 15 because Adrenaline administration performed by paramedics can be administered only to those over the age of 15.

A total of 629,471 OHCA cases were registered and 107,669 cases met the inclusion criteria. We divided them into three groups: BCPR with dispatch assistance (37,269 cases [35%]; DA-BCPR group), BCPR without dispatch assistance (18,109 cases [17%]; only BCPR group), and no BCPR (52,291 cases [49%]; No-BCPR group). Furthermore, all age groups from 15 to 114 years old, and three groups (from 64 years old or younger, 65–74 years old, and 75 years old or older) were stratified by age and compared.

### Study end point

The primary end point was the cerebral neurological outcome defined as CPC scores of 1−2. The secondary end point was the initial ECG waveform upon EMS contact, and defined as shockable rhythm (ventricular fibrillation and pulseless ventricular tachycardia).

### Statistical analysis

We stratified the time from the 119-call to EMS contact with patient by tertiles (7 min, 8−10 min, 11−20 min), and evaluated the survival and shockable rhythm rates including the correlation between DA-BCPR and time interval between the 119 call and EMS contact. Multivariable logistic regression was used to calculate the adjusted odds ratio (AOR) and 95% confidence interval (95% CI).

The following potential confounding factors in the relationship of DA-BCPR and CPC 1−2 were adapted into the regression model: age, sex, year of occurrence, bystander type (family or not), initial ECG rhythm, airway device (bag-valve-mark ventilation, endotracheal intubation, oesophageal obturator airway), adrenaline administration, time between EMS contact with the patient and hospital arrival. The following potential confounding factors in the relationship of DA-BCPR to shockable rhythm state were adapted into the regression model: age, sex, year of occurrence, and bystander type (family or not).

The influence of EMS arrival on ECG rhythm has been reported by previous studies.^19^ We also evaluated how DA-BCPR affects the ECG rhythm via sub-analysis.

During multivariable logistic regression, we ensured linearity of the continuous variables with no multicollinearity or overfitting. We used JMP Pro version 13.0.0 (SAS Institute Inc., Cary, NC, USA) for analysis.

## Results

### Patient background

A total of 629,471 OHCA patients were registered and 107,669 patients who met the inclusion criteria were included in the study. They were distributed into three groups according to the BCPR status: 37,269 (35%), 18,109 (17%), and 52,291 (49%) patients in the DA-BCPR, Only-BCPR, and No-BCPR group, respectively.

We stratified the time from 119-call to EMS contact with patient into 7 min or less (38,696 cases), 8−10 min (41,215 cases), and 11−20 min (25,982 cases). There were 1776 Missing value (1.6%).

Family members helped as bystanders more frequently in the Only-BCPR group (74.4%), followed by the DA-BCPR group (63.0%) and No-BCPR group (29.6%).

The mean time from 119-call to EMS contact was as follows: 9 min (7−11) in the DA-BCPR group, 9 min (7−11) in the Only-BCPR group, and 8 min (7−10) in the No-BCPR group. The mean time from EMS contact to hospital arrival was as follows: 23 min (17−29 min) in the DA-BCPR group, 23 min (17−30 min) in the Only-BCPR group, and 24 min (18−31) in the No-BCPR group. Table 1 shows that the DA-BCPR group had a higher rate of ROSC achievement before hospital arrival (22.1%), one-month survival (17.2%), and CPC 1−2 (13.3%).

**Table 1:** Patient background.

	DA-BCPR n = 37,519	Only-BCPR n = 18,220	No-BCPR n = 52,492
Age, mean(SD)	76.0(15.3)	75.7(16.2)	75.6(14.0)
Male,№(％)	22,077(59.2)	10,367(57.2)	32,883(62.9)
Bystander (family),No.(％)	23,497(63.0)	5369(29.6)	38,894(74.4)
BCPR,No.(％)			
Chest compression Only	24,635(66.1)	10,835(60.0)	0(0.0)
Chest compression＋artificial breathing	5259(14.1)	4518(24.49)	0(0.0)
PAD	1672(4.5)	2067(11.4)	0(0.0)
Initial ECG waveform,№(％)			
VF/VT	8118(21.8)	3511(19.4)	9165(17.5)
PEA	10,166(27.3)	5078(28.0)	18,430(35.2)
Asystole	17,226(46.2)	7643(42.2)	23,884(45.7)
Others	1759(4.7)	1877(10.4)	812(1.6)
Defibrillation by EMS, №(％)	9579(25.7)	4199(23.2)	11,776(22.5)
Adrenaline administration by EMS,№(％)	10,132(27.2)	3702(20.4)	13,798(26.4)
Advanced airway management use by EMS, №(％)	17,334(46.5)	6635(36.6)	23,642(45.2)
The 119-call to the EMS contact, median(IQR), min	9(7−11)	9(7−11)	8(7−10)
The EMS contact to the hospital arrival, median (IQR), min	23(17−29)	23(17−30)	24(18−31)
ROSC achieveemnt before hospital arrival, №(％)	6607(17.7)	3999(22.1)	6867(13.1)
One-month survival, №(％)	4828(13.1)	3113(17.2)	4242(8.1)
CPC 1−2, №(％)	3390(9.1)	2401(13.3)	2348(4.5)

DA,dispatcher-assisted; BCPR, bystander cardiopulmonary resuscitation; VF, ventricular fibrillation; VT, ventricular tachycardia; PEA, pulseless electrical activity; ROSC, return of spontaneous circulation; IQR, interquartile range.

#### Relationship between the contact time and favourable cerebral function

The one-month favourable cerebral function is affected by the time taken to reach the patient (Table 2). The Only-BCPR group demonstrated a higher ratio in all time categories; within 7 min (17.4%), 8−10 min (12.0%), and 11−20 min (9.2%). In the No-BCPR group, in all age-groups, there was no difference in the survival rate by the time duration (AOR, 1.56 [CI, 1.43−1.71], 2.01 [1.80−2.24], 1.82 [1.52−2.19] for >7 min, 8−10 min, and 11−20 min, respectively; Table 2). In the Only-BCPR group, there was no difference in the one-month favourable cerebral function based on the time taken to reach the patient (AOR, 0.97 [0.87−1.08], 0.99 [0.88−1.12], 0.89 [0.75−1.06] for >7 min, 8–10 min, and 11–20 min, respectively; Table 2).

**Table 2:** The one-month favorable cerebral function changes with time to reach the patient^a^.

	7 min or less			8−10 min			11−20 min		
	ｎ(%)	COR(95%CI)	AOR(95%CI)^b^	ｎ(%)	COR(95%CI)	AOR(95%CI)^b^	ｎ(%)	COR(95%CI)	AOR(95%CI)^b^
Reference of Non-BCPR									
DA-BCPR	1491(12.1)	1.77(1.64−1.91)	1.56(1.43−1.71)	1339(9.1)	2.82(2.56−3.10)	2.01(1.80−2.24)	537(5.7)	3.12(2.67−3.66)	1.82(1.52−2.19)
Only-BCPR	1113(17.4)	2.70(2.49−2.94)	1.61(1.44−1.79)	835(12.0)	4.00(3.60−4.44)	2.02(1.77−2.31)	422(9.2)	5.23(4.43−6.17)	2.05(1.67−2.52)
No-BCPR	1441(7.2)	reference	reference	674(3.4)	reference	reference	226(1.9)	reference	reference
Reference of NDA-BCPR									
DA-BCPR	–	0.65(0.60−0.71)	0.97(0.87−1.08)	–	0.71(0.64−0.77)	0.99(0.88−1.12)	–	0.60(0.52−0.68)	0.89(0.75−1.06)
Only-BCPR	–	reference	reference	–	reference	reference	–	reference	reference
No-BCPR	–	0.37(0.34−0.40)	0.62(0.56−0.69)	–	0.25(0.23−0.28)	0.50(0.43−0.57)	–	0.19(0.16−0.23)	0.49(0.40−0.60)

COR, crude odds ratio; AOR, adjusted odds ratio; 95%CI, 95% confidence interval; DA, dispatcher-assisted; BCPR, bystander cardiopulmonary resuscitation.

a 1776 cases (1.6%) of the missing time data (time from 119-call to the EMS contact) was excluded.

b The following confounding factor was corrected by Multivariable logistic regression; age [one year increasing], sex, occurrence year, bystander type [family or not], initial ECG waveform, airwat device airway device (BVM, ET intubation, esophageal obturator airway), adrenaline administration, time between EMS contacts to patient and the hospital arrival [one minute increasing].

The survival rate showed an association with the time taken from EMS contact to patient arrival at hospital as well as the age (Table 3). There was no difference between the no-BCPR group and the BCPR group in all categories: under 64 years old, 65−74 years, and over 75 years old.

**Table 3:** The survival rate depends on time duration from EMS contact to patient arrival as well as by age^a^.

	AOR(95%CI)^b^		
	7 min or less	8−10 min	11−20 min
Under 64 years old			
DA-BCPR	1.79(1.55−2.06)	2.41(2.05−2.83)	2.26(1.71−2.99)
Only-BCPR	1.91(1.62−2.25)	2.23(1.84−2.72)	2.60(1.90−3.55)
No-BCPR	reference	reference	reference
65−74 years old			
DA-BCPR	1.94(1.63−2.32)	1.78(1.44−2.21)	1.93(1.35−2.75)
Only-BCPR	1.78(1.43−2.21)	2.12(1.64−2.74)	1.92(1.26−2.91)
No-BCPR	reference	reference	reference
Over 75 years old			
DA-BCPR	1.13(0.96−1.34)	1.76(1.42−2.19)	1.55(1.12−2.13)
Only-BCPR	1.21(1.00−1.47)	1.82(1.41−2.35)	1.85(1.29−2.66)
No-BCPR	reference	reference	reference

AOR, adjusted odds ratio; 95%CI, 95% confidence interval; DA, dispatcher-assisted; BCPR, bystander cardiopulmonary resuscitation.

a 1776 cases (1.6%) of the missing time data (time from 119-call to the EMS contact) was excluded.

b The following confounding factor was corrected by Multivariable logistic regression; age [one year increasing], sex, occurrence year, bystander type [family or not], initial ECG waveform, airwat device airway device (BVM, ET intubation, esophageal obturator airway), adrenaline administration, time between EMS contacts to patient and the hospital arrival.

#### Shockable rhythm rate upon EMS arrival

Table 4 compares the shockable rhythm rates as a function of the time taken to reach the patient. The rates were higher in the Only-BCPR group: 26.0% within 7 min, 23.3% at 8−10 min, and 19.7% at 11−20 min.

**Table 4:** The shockable rate upon EMS arrival in all ages^a^.

	7 min or less			8−10 min			11−20 min		
	ｎ(%)	COR(95%CI)	AOR(95%CI)^b^	ｎ(%)	COR(95%CI)	AOR(95%CI)^b^	ｎ(%)	COR(95%CI)	AOR(95%CI)^b^
Reference of Non-BCPR									
DA-BCPR	2961(25.4)	1.16(1.10−1.23)	1.30(1.23−1.38)	3276(23.3)	1.49(1.41−1.57)	1.60(1.51−1.70)	1786(19.7)	1.81(1.68−1.96)	1.90(1.75−2.06)
Only-BCPR	1459(26.0)	1.19(1.12−1.28)	1.16(1.08−1.26)	1257(20.7)	1.29(1.19−1.38)	1.36(1.25−1.48)	750(17.8)	1.60(1.46−1.77)	1.67(1.50−1.86)
No-BCPR	4444(22.7)	reference	reference	3290(16.9)	reference	reference	1401(11.9)	reference	reference
Reference of NDA-BCPR									
DA-BCPR	–	0.97(0.90−1.05)	1.12(1.03−1.21)	–	1.16(1.08−1.25)	1.18(1.08−1.28)	–	1.13(1.03−1.24)	1.13(1.02−1.26)
Only-BCPR	–	reference	reference	–	reference	reference	–	reference	reference
No-BCPR	–	0.84(0.78−0.90)	0.86(0.80−0.93)	–	0.78(0.73−0.84)	0.73(0.68−0.80)	–	0.62(0.57−0.69)	0.60(0.54−0.68)

COR, crude odds ratio; AOR, adjusted odds ratio; 95%CI, 95% confidence interval; DA, dispatcher-assisted; BCPR, bystander cardiopulmonary resuscitation.

a 1776 cases (1.6%) of the missing time data (time from 119-call to the EMS contact) was excluded.

b The following confounding factor was corrected by Multivariable logistic regression; age [one year increasing], sex, occurrence year, bystander type [family or not].

In all cases, when the time interval from 119-call to EMS contact was within 7 min, the AOR was 1.30 (1.23−1.38) in the DA-BCPR and 1.19 (1.12−1.28) in the Only-BCPR groups. For 8−10 min, the AOR was 1.60 (1.51−1.70) in the DA-BCPR group and 1.36 (1.25−1.48) in the Only-BCPR group. For the 11−20 min interval, AOR was 1.90 (1.75−2.06) in the DA-BCPR group and 1.67 (1.50−1.86) in the Only-BCPR group (Table 5).

**Table 5:** The shockable rate upon EMS arrival by age^a^.

	AOR(95%CI)^b^		
	7 min or less	8−10 min	11−20 min
Under 64 years old			
DA-BCPR	1.53(1.37−1.70)	1.99(1.80−2.20)	2.48(2.16−2.85)
Only-BCPR	1.38(1.20−1.58)	1.62(1.41−1.86)	1.88(1.58−2.25)
No-BCPR	reference	reference	reference
65−74 years old			
DA-BCPR	1.30(1.23−1.38)	1.60(1.51−1.70)	1.90(1.75−2.06)
Only-BCPR	1.16(1.08−1.26)	1.36(1.25−1.48)	1.70(1.50−1.86)
No-BCPR	reference	reference	reference
Over 75 years old			
DA-BCPR	1.18(1.08−1.30)	1.35(1.23−1.48)	1.64(1.44−1.87)
Only-BCPR	1.01(0.89−1.15)	1.25(1.10−1.43)	1.62(1.36−1.94)
No-BCPR	reference	reference	reference

AOR, adjusted odds ratio; 95%CI, 95% confidence interval; DA, dispatcher-assisted; BCPR, bystander cardiopulmonary resuscitation.

a 1776 cases (1.6%) of the missing time data (time from 119-call to the EMS contact) was excluded.

b The following confounding factor was corrected by Multivariable logistic regression; age [one year increasing], sex, occurrence year, bystander type [family or not].

In the under 64 years old category, the DA-BCPR group and Only-BCPR group showed AOR of 1.53 (1.37−1.70) and 1.38 (1.20−1.58) within 7 min, 1.99 (1.80−2.20) and 1.62 (1.41−1.86) between 8−10 min, and 2.48 (2.16−2.85) and 1.88 (1.58−2.25) between 11−20 min, respectively. In the 65−74 years category, the DA-BCPR group and Only-BCPR group showed AOR of 1.30 (1.23−1.38) and 1.16 (1.08−1.26) within 7 min, 1.60 (1.51−1.70) and 1.36 (1.25−1.48) between 8−10 min, and 1.90 (1.75−2.06) and 1.70 (1.25–1.86) between 11−20 min, respectively. In the over 75 years category, the DA-BCPR group and Only-BCPR group showed AOR of 1.18 (1.08−1.30) and 1.01 (0.89−1.15) within 7 min, 1.35 (1.23−1.48) and 1.25 (1.10−1.43) between 8−10 min, and 1.64 (1.44−1.87) and 1.62 (1.36−1.94) between 11−20 min, respectively.

## Discussion

### Major findings

Bystander witnesses cardiogenic OHCA cases were analysed in this study. On comparing the DA-BCPR and No-BCPR groups, the survival rates were increased regardless of the time course. Age-categorized analyses were also similar in terms of the time course and survival rate. However, compared to the Only-BCPR group, the DA-BCPR group showed a greater correlation with the shockable rhythm rate improvement regardless of the time course.

### Previous studies

Dispatch assistance has been described worldwide, and BCPR with dispatch assistance demonstrates good post-OHCA outcomes, such as improving the survival rate, similar to BCPR without dispatch assistance.^26^, ^27^, ^28^, ^29^ In a study investigating the correlation between DA-BCPR and survival rate, Rea et al. found that reducing the time from patient collapse to initiation of CPR improved the survival rate in both DA-BCPR and Only-BCPR groups compared to the No-BCPR group.^22^ Hagihara et al. analysed the effectiveness of DA-BCPR depending on the time from patient collapse to initiation of BCPR; the DA-BCPR group tended to show decreased survival rates when the patient-contact time got longer.^30^ Hagihara et al. also reported that dispatch assistance can decrease the quality of BCPR.^30^ Our study had a different perspective from this study because we examined the effectiveness of DA-BCPR by comparing with the Only-BCPR and No-BCPR groups.

In 2019, Lee et al. studied the effect of DA-BCPR on cardiogenic OHCA with respect to defibrillation and ROSC. They reported that BCPR, with or without DA, was associated with defibrillation and ROSC, and led to improvement in the survival of OHCA patients until discharge, compared to cases where BCPR was not provided.^31^ However, unlike Lee et al. In our study, our study compared the DA-BCPR group and No-BCPR group, and showed that DA-BCPR is effective regardless of the arrival time of EMS and age.

### DA-BCPR group and shockable rhythm rate

Another notable finding of this study is that the DA-BCPR group was associated with an improvement in the shockable rhythm rate regardless of the passage of time compared to the BCPR group. Takahashi et al. reported that improvement in the shockable rhythm rate upon EMS arrival was associated with BCPR.^21^ It is considered that DA-BCPR improves the coronary perfusion pressure, resulting in an improvement in the shockable rhythm rate and increased defibrillation rate. Consequently, this can lead to an improvement in the survival rate and neurological prognosis.

#### Effects of DA-BCPR

A review of the International Guidelines 2000 for CPR reported that preventing deterioration from ventricular fibrillation to asystole before arrival of EMS increases the success rate of defibrillation, contributes to the maintenance of cardiac and brain function, and improves survival.^12^ However, this period involves CPR with combined ventilation and chest compressions. In Japan, when implementing DA, CPR was unified throughout Japan to include chest compressions alone, as recommended in the Guideline 2010. Our study investigates this period. Accordingly, it was inferred that CPR with chest compressions alone has the same effect as CPR with combined ventilation and chest compressions.

In Japan, the average time from the 119 call to the arrival of EMS at the scene is 8.6 min nationwide, 7.1 min on average in the shortest prefecture, and 10.9 min on average in the longest prefecture,^6^ However, many OHCAs occur at home.^32^, ^33^, ^34^ In some cases, there may be only one bystander, and dispatchers rarely direct the caller to get an AED to a location other than his home or perform defibrillation. Even if it takes time for EMS to arrive at a site due to local circumstances, actively promoting DA-BCPR can improve the shockable rhythm rate and survival rate. In other words, oral guidance that encourages BCPR implementation is an effective measure to improve the survival rate, especially if the dispatcher determines that the patient is in cardiac arrest. BCPR combined with AED can play an important role in improving the clinical survival rate after cardiogenic OHCA.^7^, ^8^, ^9^, ^10^, ^11^, ^12^ In the future, it is necessary to quickly contact first responders scattered around the area and build a community AED system to improve availability of AEDs.

## Limitations

Our study has several limitations. Since this was an observational study, we were unable to manage the unmeasured and unknown cofounders. Additionally, we did not collect any in-hospital treatment data that may correlate with cerebral function post-OHCA.^35^, ^36^

Data regarding the reason why the bystanders did not perform DA-BCPR were not collected. Although the protocol of dispatch assistance is guided by the FDMA, it may be different depending on the local MC or fire department. Some areas may recommend a combination of chest compressions and artificial breathing; thus, we could not control such interactions. Since this study only analysed witnessed cardiogenic OHCA cases, we cannot generalise the findings to unwitnessed or non-cardiogenic cases.

## Conclusion

We analysed DA-BCPR in witnessed cardiogenic OHCA cases and found favourable cerebral function outcomes, as well as improvement in the shockable rhythm rate that can influence long-term outcomes. DA-BCPR was associated with better survival of brain function regardless of the EMS arrival time when compared to the No-BCPR group. It was also suggested that if there was no AED near the cardiac arrest patient and BCPR with DA was performed, the shockable rhythm rate could improve even if there was a difference in the arrival time of EMS.

It is important for dispatchers to promote BCPR regardless of the arrival time at the EMS site in order to improve the prognosis of brain function and the shockable rhythm rate.

## Declaration of interests

The authors declare that they have no known competing financial interests or personal relationships that could have appeared to influence the work reported in this paper.

## CRediT authorship contribution statement

**Minoru Kayanuma:** Conceptualization, Methodology, Project administration, Formal analysis, Data curation, Writing - original draft, Visualization. **Ryo Sagisaka:** Conceptualization, Methodology, Data curation, Validation, Writing - review & editing. **Hideharu Tanaka:** Writing - review & editing, Supervision. **Shota Tanaka:** Writing - review & editing.
